# New agents that target senescent cells: the flavone, fisetin, and the BCL-X_L_ inhibitors, A1331852 and A1155463

**DOI:** 10.18632/aging.101202

**Published:** 2017-03-08

**Authors:** Yi Zhu, Ewald J. Doornebal, Tamar Pirtskhalava, Nino Giorgadze, Mark Wentworth, Heike Fuhrmann-Stroissnigg, Laura J. Niedernhofer, Paul D. Robbins, Tamara Tchkonia, James L. Kirkland

**Affiliations:** ^1^ Robert and Arlene Kogod Center on Aging, Mayo Clinic, Rochester, MN 55905, USA; ^2^ Faculty of Science and Engineering, University of Groningen, University Medical Center Groningen, Groningen, Netherlands; ^3^ Office of Research Regulatory Support, Mayo Clinic, Rochester, MN 55905, USA; ^4^ Department of Molecular Medicine and the Center on Aging, The Scripps Research Institute, Jupiter, FL 33458, USA

**Keywords:** senolytics, aging, adipose-derived stem cells, preadipocytes, apoptosis, flavonoids, BCL-X_L_ inhibitors

## Abstract

Senescent cells accumulate with aging and at sites of pathology in multiple chronic diseases. Senolytics are drugs that selectively promote apoptosis of senescent cells by temporarily disabling the pro-survival pathways that enable senescent cells to resist the pro-apoptotic, pro-inflammatory factors that they themselves secrete. Reducing senescent cell burden by genetic approaches or by administering senolytics delays or alleviates multiple age- and disease-related adverse phenotypes in preclinical models. Reported senolytics include dasatinib, quercetin, navitoclax (ABT263), and piperlongumine. Here we report that fisetin, a naturally-occurring flavone with low toxicity, and A1331852 and A1155463, selective BCL-X_L_ inhibitors that may have less hematological toxicity than the less specific BCL-2 family inhibitor navitoclax, are senolytic. Fisetin selectively induces apoptosis in senescent but not proliferating human umbilical vein endothelial cells (HUVECs). It is not senolytic in senescent IMR90 cells, a human lung fibroblast strain, or primary human preadipocytes. A1331852 and A1155463 are senolytic in HUVECs and IMR90 cells, but not preadipocytes. These agents may be better candidates for eventual translation into clinical interventions than some existing senolytics, such as navitoclax, which is associated with hematological toxicity.

## INTRODUCTION

Senescent cells accumulate in numerous tissues with aging and at sites of pathogenesis of multiple chronic diseases [1, 2]. Small numbers of senescent cells can cause extensive local and systemic dysfunction due to their pro-inflammatory senescence-associated secretory phenotype (SASP) [3]. For example, transplanting only 2X10^5^ senescent ear chondroblasts or preadipocytes around knee joints induces osteoarthritis in mice, while injecting similar numbers of non-senescent cells does not [4]. Clearing senescent cells by activating a drug-inducible “suicide” gene in progeroid or naturally-aged mice alleviates a range of age- and disease-related phenotypes, including sarcopenia, frailty, cataracts, adipose tissue dysfunction, insulin resistance, and vascular hyporeactivity [5-7].

To decrease the burden of senescent cells in non-genetically-modified individuals, we used a hypothesis-driven approach to identify senolytic compounds, which preferentially induce apoptosis in senescent rather than normal cells [8, 9]. Our approach was based on the observation that senescent cells are resistant to apoptosis [10]. This suggested that senescent cells either have reduced engagement of pro-apoptotic pathways that serve to protect them from their own pro-apoptotic SASP or they have up-regulated pro-survival pathways [8]. We demonstrated the latter to be the case and identified senescence-associated pro-survival pathways based on expression profiling of senescent *vs*. non-senescent cells. We confirmed the requirement of these pathways for survival of senescent but not non-senescent cells by RNA interference. These pathways included pro-survival networks related to PI3K/ AKT, p53/ p21/ serpines, dependence receptor/ tyrosine kinases, and BCL-2/BCL-X_L_, among others.

**Figure 1:**
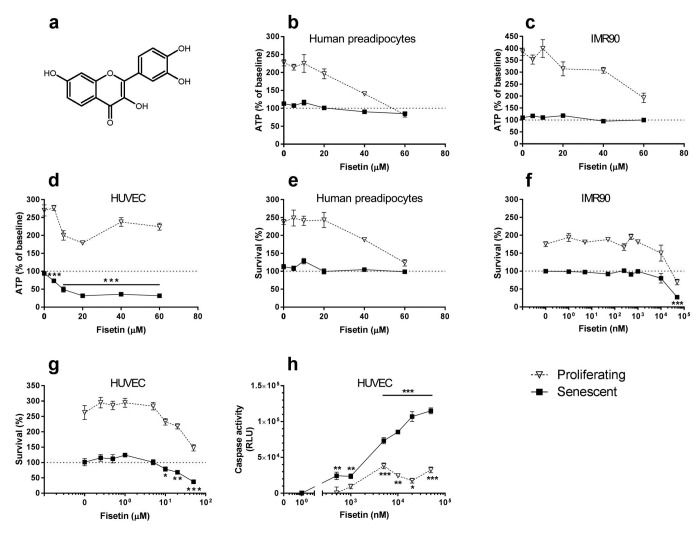
Fisetin targets senescent cells (**a**) Structure of fisetin. (**b**-**d**) Fisetin is more effective in reducing viability (ATPLite) of senescent HUVECs than IMR90 cells or primary human preadipocytes. Proliferating or senescent cells were exposed to different concentrations of fisetin for 3 days. The red lines denote ATPLite intensities on day 0 of senescent and non-senescent cells, both set to 100%. HUVEC and IMR90 data are means±SEM of 5 replicates at each drug concentration. Preadipocyte data are means±SEM of 5 replicates from each of 4 different subjects at each concentration. (**e**-**g**) Fisetin selectively reduces senescent but not proliferating HUVECs and IMR90 cell numbers (crystal violet). The red lines denote cell numbers at plating on day 0 of senescent and non-senescent cells, both set to 100%. HUVEC and IMR90 data are means±SEM of 5 replicates at each drug concentration. Preadipocyte data are means±SEM, 5 replicates from each of 4 different subjects at each concentration. (**h**) Fisetin induces apoptosis in senescent HUVECs. HUVECs were treated with fisetin for 12h and then caspases-3&7 were assayed using a luminescent substrate. Fisetin (500 nM) induced apoptosis in senescent cells by caspase 3/7 assay. For all figures: * = P<0.05; ** = P<0.01; *** = P<0.001 by one-way ANOVA (caspase activities by 2-way ANOVA). Bars with asterisks indicate differences between senescent cells following drug exposure compared to day 0.

We tested drugs that target these pro-survival pathways. We initially reported that the dependence receptor/ tyrosine kinase inhibitor, dasatinib (D) and the flavonoid, quercetin (Q), are senolytic *in vitro* and *in vivo*. D and Q induced apoptosis in senescent primary human preadipocytes and HUVECs, respectively. Combining D+Q broadened the range of senescent cells targeted, and, in some instances, proved synergistic in some types of senescent cells [8]. D+Q alleviated cardio-vascular, frailty-related, osteoporotic, neurological, radiation-induced, and other phenotypes and disorders in chronologically aged, progeroid, and high fat-fed atherosclerosis-prone mice, consistent with our observations in mice from which senescent cells had been removed by inducing the suicide gene in transgenic INK-ATTAC mice [5-8]. Expanding upon our findings with Q, we tested if the related flavonoid, fisetin (Fig. 1A), is senolytic. Fisetin is widely available as a nutritional supplement and has a highly favorable side-effect profile.

**Figure 2:**
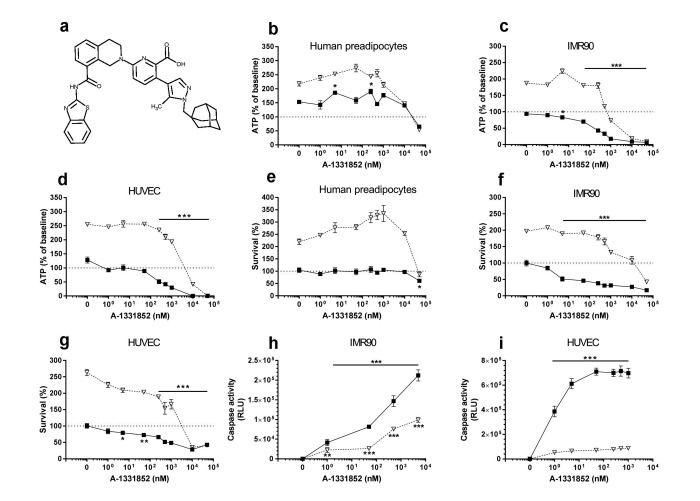
A1331852 targets senescent cells (**a**) Structure of A1331852. (**b**-**d**) A1331852 is more effective in reducing viability (ATPLite) of senescent HUVECs and IMR90 cells than primary human preadipocytes. Proliferating or senescent cells were exposed to different concentrations of A1331852 for 3 days. The red lines denote ATPLite intensities on day 0 of senescent and non-senescent cells, both set to 100%. HUVEC and IMR90 data are means±SEM of 5 replicates at each drug concentration. Preadipocyte data are means±SEM of 5 replicates from each of 4 different subjects at each concentration. (**e**-**g**) A1331852 selectively reduces senescent but not proliferating HUVECs and IMR90 cell numbers (crystal violet). The red lines denote cell numbers at plating on day 0 of senescent and non-senescent cells, both set to 100%. HUVEC and IMR90 data are means±SEM of 5 replicates at each drug concentration. Preadipocyte data are means±SEM of 5 replicates from each of 4 different subjects at each concentration. (**h**-**i**) A1331852 induces apoptosis in senescent HUVECs and IMR90 cells. HUVECs were treated with A1331852 for 12h and then caspases-3&7 were assayed using a luminescent substrate. A1331852 (1 nM) induced apoptosis in senescent cells by caspase 3/7 assay.

**Figure 3:**
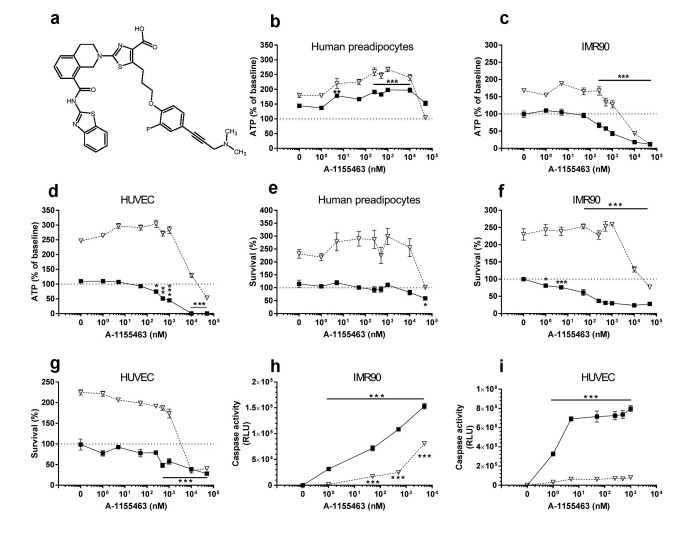
A1155463 targets senescent cells (**a**) Structure of A1155463. (**b**-**d**) A1155463 is more effective in reducing viability (ATPLite) of senescent HUVECs and IMR90 cells than primary human preadipocytes. Proliferating or senescent cells were exposed to different concentrations of A1155463 for 3 days. The red lines denote ATPLite intensities on day 0 of senescent and non-senescent cells, both set to 100%. HUVEC and IMR90 data are means±SEM of 5 replicates at each drug concentration. Preadipocyte data are means±SEM of 5 replicates from each of 4 different subjects at each concentration. (**e**-**g**) A1155463 selectively reduces senescent but not proliferating HUVECs and IMR90 cell numbers (crystal violet). The red lines denote cell numbers at plating on day 0 of senescent and non-senescent cells, both set to 100%. HUVEC and IMR90 data are means±SEM of 5 replicates at each drug concentration. Preadipocyte data are means±SEM of 5 replicates from each of 4 different subjects at each concentration. (**h**-**i**) A1155463 induces apoptosis in senescent HUVECs and IMR90 cells. HUVECs were treated with A1155463 for 12h and then caspases-3&7 were assayed using a luminescent substrate. A1155463 (1 nM ) induced apoptosis in senescent cells by caspase 3/7 activity assay.

Based on our earlier hypothesis-driven identification of senolytic drugs and identification of the BCL-2 pro-survival pathway as one of the “Achilles' heels” of senescent cells [8], we and others simultaneously reported that the BCL-2/ BCL-W/ BCL-X_L_ inhibitor, navitoclax (ABT263; N), is senolytic [11, 12]. Like D and Q, N is senescent cell type-specific, being effective in inducing apoptosis in HUVECs but not human preadipocytes. This is consistent with our initial report, in which we observed that RNA interference against BCL-X_L_ is senolytic in HUVECs, but not primary human preadipocytes [8]. We also found that the related BCL-2 family inhibitor, TW-37, is not senolytic. TW-37, unlike N, does not target BCL-X_L_. Others confirmed that N targets senescent cells, but Bcl-2 family inhibitors that do not target BCL-X_L_ are not senolytic [12, 13]. We therefore tested if the relatively specific BCL-X_L_ inhibitors, A1331852 and A1155463 (Figs. 2A & 3A, respectively) [14], are senolytic. Unlike N, these agents do not target BCL-2. Consequently, A1331852 or A1155463 may cause less BCL-2-induced neutrophil toxicity, a serious side-effect of N [14].

## RESULTS

To assess if fisetin is senolytic, we cultured primary human preadipocytes, HUVECs, and IMR90 cells and exposed these cells to 10Gy radiation (senescent cells) or sham-irradiated them (control cells). Fisetin (Fig. 1a), like its analog quercetin [8], selectively reduced viability (ATPLite; Fig. 1b-d) and numbers (crystal violet; Fig. 1e-g) of senescent HUVECs, but not IMR90 cells or primary human preadipocytes. It induced apoptosis in senescent HUVECs, confirmed by caspase3/7 activity assay (Fig. 1h). The concentrations at which fisetin caused caspase activity to increase, viability as assessed by ATPLite to decrease, and cell numbers as assessed by crystal violet to decrease to below those levels at the time the cells had originally been plated were: 0.5 μM, 5 μM, and 10 μM, respectively. The different concentration thresholds may reflect activation of caspases at lower concentrations or earlier times than those required to interfere with viability and cell death.

The BCL-X_L_ inhibitors, A1331852 (Fig. 2a) and A1155463 (Fig. 3a), selectively reduced viability (Figs. 2b-d & 3b-d, respectively) and survival (Figs. 2e-g & 3e-g, respectively) of senescent HUVECs and IMR90 cells, but not senescent preadipocytes. A1331852 and A1155463 increased ATP in proliferating preadipocytes (Figs. 2b &3b). As with fisetin, apoptosis was induced by A1331852 and A1155463, as demonstrated by caspase3/7 activity assays (Figs. 2h-i & 3h-i, respectively). Also as with fisetin, concentrations at which A1331852 and A1155463 began to affect caspase < ATPLite < crystal violet. The observation that senescent HUVECs were more sensitive to the BCL-X_L_ inhibitors than human preadipocytes is consistent with our findings using RNA interference in the first article reporting senolytics [8].

## DISCUSSION

We found that fisetin and the BCL-X_L_ inhibitors, A1331852 and A1155463, are senolytic *in vitro*, inducing apoptosis in senescent, but not non-senescent HUVECs. This adds three new agents to the emerging repertoire of senolytics reported since early 2015, which currently includes D, Q, N, and piperlongumine [8, 11, 12, 15].

Fisetin (3,3′,4′,7-tetrahydroxyflavone) is a member of the flavonoid family, a group of naturally occurring polyphenolic compounds [16]. It is present in low concentrations in many fruits and vegetables such as apples, persimmon, grapes, onions, and cucumbers, with the highest concentration found in strawberries (160μg/g) [16]. The average dietary intake of naturally occurring fisetin was approximately 0.4 mg/day in a Japanese study [17]. Due to it's being hydrophobic, fisetin penetrates cell membranes and accumulates within cells to exert antioxidant effects [18]. Other promising biological activities of fisetin include anti-hyperglycemic, anti-hyperlipidemic, anti-inflammatory, neurotrophic, and anti-carcinogenic effects [16, 19-26]. Fisetin promotes apoptosis in human breast cancer MCF-7 cells by activating caspases-7,8,&9 without causing apoptosis in non-tumorigenic cells [25]. Fisetin has a plasma terminal half-life of just over 3 hours in mice, with its metabolites being excreted in feces and urine. It alleviates dysfunction in animal models of chronic disease, including diabetic kidney disease and acute kidney injury [19, 20, 27-29], attributes consistent with those expected from a senolytic agent [9]. Here we demonstrate that fisetin is indeed senolytic in senescent HUVECs, but not in senescent IMR-90 cells or human preadipocytes. Interestingly, the fisetin concentrations achieved in a mouse study without causing toxicity (2.7- 349.4 μM) [30] are similar to and even higher than those we found to be senolytic in cultured HUVECs.

The intrinsic mitochondrial apoptosis pathway is inhibited by the BCL-2 (B-cell lymphoma-2) protein family. BCL-2, BCL-X_L_ (BCL-2 related protein, long isoform), and MCL-1 (myeloid cell leukemia-1) bind the BH3 motifs of pro-apoptotic proteins, including BIM (BCL-2 interacting mediator of cell death), BAK (BCL-2 antagonist/killer), and BAX (BCL-2 associated X protein). N, which targets the BCL-2 family members, BCL-2, BCL-W, and BCL-X_L_, is senolytic [11, 12]. However, drugging of BCL-2-related proteins can involve serious side-effects, including depletion of platelets and neutrophils, as observed with N [31].

Among the BCL-2-related proteins, BCL-X_L_ is a particularly attractive therapeutic target, since it is required for survival of senescent HUVECs, as demonstrated in our RNA interference studies [8]. Although the BCL-2 inhibitors have a number of side-effects, A1331852 and A1155463 are relatively selective BCL-X_L_ inhibitors [14] and appear less likely to cause neutropenia than N, making them potentially better candidates for eventual translation into clinical applications. A1331852 and A1155463 are senolytic in HUVECs and IMR-90 cells but not primary human preadipocytes. We noted that these drugs increased cellular ATP levels significantly in senescent human preadipocytes, but not HUVECs, through an as yet unknown mechanism.

We predict many more senolytic drugs will appear at an accelerating pace over the next few years. Initially, most are likely to be based on re-purposed drugs or natural products. Increasingly, new senolytics will likely be derived using medicinal chemical approaches based on optimizing properties of the repurposed agents. Consistent with this, it appears that small changes in the senolytic drugs already discovered can interfere with senolytic activity, such as in the case of D *vs*. imatinib, with the latter not being senolytic, or N *vs*. the closely-related agent, TW-37. Conversely, we speculate that small structural changes to repurposed senolytic drugs could enhance senolytic activity, with increases in the percent and range of types of senescent cells eliminated, as well as better stability, bioavailability, and side-effect profiles.

Many more senolytics will likely be discovered in the near future, with some targeting the senolytic pathways we discovered using bioinformatics coupled with RNA interference approaches. These include dependence receptor/ tyrosine kinase- (*e.g*., D), PI3K/ AKT/ metabolic- (*e.g*., Q and fisetin), BCL-2- (*e.g*., N, A1331852, and A1155463), p53/ p21/ serpine (PAI-1&2)-, and HIF-1α-related pathways [8]. We also predict that many or most new agents will target particular types of senescent cells, such as senescent cells originating from different cell types or varying in the mechanisms through which senescence was induced. Consistent with this, fisetin, a flavonoid that is related to Q, was senolytic for senescent HUVECs but not preadipocytes, as was the case for Q [8]. Furthermore, we previously found by RNA interference that BCL-X_L_ is needed for survival of senescent HUVECs, but not human preadipocytes. Consistent with this observation is the fact that neither A1331852 nor A1155463 are senolytic for human preadipocytes. This cell type specificity could mean that particular senolytic drugs or combinations will be more effective for some age- or senescence-related indications than others. For example, it could be speculated that combining fisetin, A1331852, or A1155463 with D or a related senolytic active against senescent preadipocytes might be more effective than fisetin, A1331852, or A1155463 alone for obesity-related indications, a point that needs to be studied in appropriate pre-clinical models.

Some side effects of senolytic drugs may extend across the class. These include delays in skin wound healing, since senescent cells may facilitate certain phases of wound resolution and tissue repair [32]. At higher concentrations than those that cause apoptosis in senescent cells, fisetin, A1331852, and A1155463 may be cytostatic in proliferating cells. This inhibition of proliferation is not associated with apoptosis in non-senescent cells until yet higher concentrations are reached. This suggests that, like most drugs, these agents will have a concentration window over which they are effective and safe, with lower concentrations failing to be senolytic and higher concentrations likely being toxic. Other serious side effects of senolytics as a class have not become apparent so far in preclinical studies using these agents or in studies of clearance of senescent cells in transgenic mice expressing drug-inducible “suicide” proteins in their senescent cells. However, it is likely that more class-specific side effects will emerge over time. An advantage of seno-lytics over drugs that must be present continuously to be effective is that senolytics likely can be administered intermittently, reducing the opportunity for side effects to occur.

Other effects and side effects of individual senolytics are likely to be drug-specific. Fisetin has few known side effects so far [33], unlike BCL-2 inhibitors. Fisetin is metabolized by glucuronidation [30], so it could potentiate effects of warfarin, necessitating reduction in the dose of warfarin and other drugs. On balance, fisetin, D, Q, and piperlongumine appear to have strong potential for becoming orally-active senolytic agents for clinical use, or at least to become scaffolds that can be optimized using medicinal chemical approaches for use as oral agents. N and possibly A1331852 and A1155463 could see use as injected senolytic agents, allowing effective local senolytic concentrations to be achieved while reducing the likelihood of systemic side effects. A1331852 and A1155463 could be safer than N since they have less effects on neutrophil levels. Alternatively, BCL-2 inhibitors could be used systemi-cally at lower doses, possibly in combination with other senolytic drugs.

Combining senolytics with distinct, additive mechanisms of action is an attractive option that needs to be investigated further in preclinical studies. Consistent with the potential value of this approach, the combination of D+Q has broader senolytic activity than either agent alone [8] and the combination of D and A1331852 is more effective than either drug alone in inducing apoptosis in chronic myeloid leukemia cells [34]. Furthermore, combinations of senolytics with other agents that target fundamental aging mechanisms, such as 17α-estradiol, that act through mechanisms other than targeting senescent cells [35] may prove to be more effective than individual agents and could flatten side-effect profiles.

Senolytics have potential for delaying, preventing, or alleviating a number of age-related phenotypes and chronic diseases, including diabetes, osteoporosis, frailty, cardiovascular disease, pulmonary fibrosis, and cancers, among others [2, 6-8]. Clinical trials are needed to determine the safety and efficacy of these drugs before routine clinical use is considered. This includes agents that are available over the counter as nutritional supplements. Now that we have shown that fisetin, A1331852, and A1155463 are senolytic *in vitro*, studies are needed to determine if these new additions to the growing number of senolytic agents reduce senescent cell burden *in vivo* and alleviate dysfunction in aged animals and pre-clinical animal models of age- and senescence-related diseases.

## MATERIALS AND METHODS

### Isolation and cell culture of primary human preadipocytes

Abdominal subcutaneous adipose tissue for primary preadipocyte isolation was obtained during intra-abdominal surgery from 4 healthy, lean subjects undergoing surgery to donate a kidney (male; age 45.2±2.4 [mean ± SEM] years), who had given informed consent. The cells were passaged 4 population doublings. Preadipocytes are also known as adipose-derived stem cells or fat cell progenitors (for detailed discussion of nomenclature, see [36]). The protocol was approved by the Mayo Clinic Foundation Institutional Review Board for Human Research. Detailed descriptions of preadipocyte isolation and cell culture conditions are in our publications [8, 11, 37].

### Human Umbilical Vein Endothelial Cell (HUVEC) culture

Human umbilical vein endothelial cells (HUVECs) were purchased from Lonza (Lonza, Walkersville, MD) and grown in Clonetics Endothelial Cell Growth Medium-2 (EGM-2; Lonza) according to the manufac-turer's protocol.

### IMR90 cell culture

IMR90 cells were purchased from ATCC (Manassas, VA) and grown in Dulbecco's Modified Eagle's Medium with 10% fetal bovine serum according to the guide provided by ATCC.

### Induction of cellular senescence

HUVECs, IMR90 cells, or human primary preadipocytes at passage 4 were radiated with 10 Gy to induce senescence or were sham-radiated. Preadipo-cytes were senescent by 20 days after radiation, IMR90 cells after 20 days, and HUVECs after 10 days, with 90% or more cells positive for senescence-associated β-galactosidase activity and by increased SASP factor expression by ELISA (IL-6, MCP-1), as in [6, 8, 11].

### Sources of agents and addition to cultures

Fisetin powder was purchased from Sigma (cat# F 4043, St. Louis, MO). A 60 mM stock solution of fisetin in DMSO was stored at −80°C until use. This stock solution was then further diluted in DMSO and added to culture media, so that the final fisetin concentrations in culture media shown in each figure were achieved with 0.1% DMSO:medium (v/v). A1331852 and A1155463 were purchased as powders from Selleckchem (cat# S7801 and cat# S7800, respectively; Houston, TX). A 50 mM stock solution for each drug of A1331852 or A1155463 was prepared in DMSO and stored at −80°C until use This stock solution was then further diluted in DMSO and added to culture media, so that the final A1331852 or A1155463 concentrations in culture media shown in each figure were achieved with 0.1% DMSO:medium (v/v).

### ATPLite assay

Cell viability was measured using an ATPLite Kit (cat# 6016941; PerkinElmer, Waltham, MA). The assay was performed according to the manufacturer's instructions. Luminescence was read using a multi scan plate reader (Fisher, Waltham, MA).

### Crystal violet assay

Viable cell numbers were measured by staining with crystal violet. Cells were washed twice with PBS, incubated with PBS containing 4% paraformaldehyde for 15 minutes at room temperature, and then stained with 0.1% crystal violet for 30 minutes at room temperature. Cells were washed with deionized water and staining intensity was measured at λ540 using a multi scan plate reader (Fisher, Waltham, MA).

### Caspase 3/7 assay

Induction of apoptosis was measured with a Caspase-Glo® 3/7 Assay kit (Promega, Cat.# G8091, Madison, WI) 12 hours after exposing cells to different concentrations of drugs. The activity of caspase3/7 was assessed by luminescence intensity using a multi scan plate reader (Fisher, Waltham, MA).

### Statistical methods

One- and two-way ANOVA tests were conducted using Prism 7.01 (GraphPad Software Inc.; La Jolla, CA).
